# Treatment of Fungal-Infected Diabetic Wounds with Low Temperature Plasma

**DOI:** 10.3390/biomedicines10010027

**Published:** 2021-12-23

**Authors:** Kyu Young Choi, Md. Tipu Sultan, Olatunji Ajiteru, Heesun Hong, Young Jin Lee, Ji Seung Lee, Hanna Lee, Ok Joo Lee, Soon Hee Kim, Joong Seob Lee, Sung-Jin Park, James Gary Eden, Chan Hum Park

**Affiliations:** 1 Department of Otorhinolaryngology-Head and Neck Surgery, Hallym University Kangnam Sacred Heart Hospital, Seoul 07441, Korea; coolq0@hallym.or.kr; 2 Nano-Bio Regenerative Medical Institute, College of Medicine, Hallym University, 1 Hallymdaehak-gil, Chuncheon 24252, Korea; tipubge@yahoo.com (M.T.S.); ajiteruolatunji@gmail.com (O.A.); heesun181025@gmail.com (H.H.); skws789@naver.com (Y.J.L.); dbrghrl@naver.com (J.S.L.); dlgkssk1995@naver.com (H.L.); vudckd@hanmail.net (O.J.L.); soonheekkim@gmail.com (S.H.K.); 3 Department of Otorhinolaryngology-Head and Neck Surgery, Hallym University Sacred Heart Hospital, Anyang 14068, Korea; apniosio@naver.com; 4 Laboratory for Optical Physics and Engineering, Department of Electrical and Computer Engineering, University of Illinois, Urbana, IL 61801, USA; sjinparkui@gmail.com (S.-J.P.); jgeden@illinois.edu (J.G.E.); 5 Department of Otorhinolaryngology-Head and Neck Surgery, Hallym University Chuncheon Sacred Heart Hospital, Chuncheon 24253, Korea

**Keywords:** low temperature plasma, skin, wound healing, diabetes mellitus, *Candida albicans*

## Abstract

Diabetes mellitus renders patients susceptible to chronic wounds and various infections. Regarding the latter, fungal infections are of particular concern since, although they are the source of significant morbidity and mortality in immunocompromised patients, they are generally resistant to conventional treatment and a definite treatment strategy has not yet been established. Herein, we report the treatment of skin wounds in a diabetic rat model, infected by *Candida albicans*, with low temperature helium plasma generated in a hand-held atmospheric jet device. A fungal infection was induced on two dorsal skin wounds of the diabetic rats, and one wound was treated with the plasma jet whereas the other served as a control. Histological analysis revealed accelerated skin wound healing and decreased evidence of fungal infection in the plasma-treated group, as compared to the control group. Regeneration of the epidermis and dermis, collagen deposition, and neovascularization were all observed as a result of plasma treatment, but without wound contraction, scar formation or any evidence of thermal damage to the tissue. These findings demonstrate that the He plasma jet is remarkably effective in diabetic skin wounds infected by *Candida albicans*, thereby providing a promising medical treatment option for diabetes mellitus patients with skin wound and fungal infections.

## 1. Introduction

Diabetes mellitus (DM) is a metabolic disease caused by elevated levels of glucose accumulation in tissue and organs that result in the onset of neuropathy and vasculopathy. Delayed and disturbed wound healing in DM patients is a growing problem worldwide with significant morbidity and the associated economic burden [1], primarily due to the challenges in identifying treatments capable of healing wounds. Owing to their immunosuppressive state, DM patients are also predisposed to infections and fungal infections, in particular. The presence of high glucose, secretion of degradative enzymes, and the immunosuppressive state of the patient influence the balance between the host and yeast, leading to the transition of the commensal *Candida albicans* (*C. albicans)* pathogen to cause the manifestation of infection [2,3]. The development of drug resistance to *C. albicans* in diabetic patients is also a concern that can significantly impact morbidity and mortality rates [4,5]. It has been reported that 90% of fungemia cases are caused by Candida species, with the mortality rate in immunocompromised hosts known to be 40–80% [5,6]. Despite the diverse treatment strategies that have been reported including topical therapies, regenerative medicine, hyperbaric oxygen, etc. [6,7], no therapeutic method has been established as an effective strategy for the treatment of chronic wounds and infections in DM patients with the gap between the literature and practice still existing.

Over the past decade, low temperature plasma has been demonstrated to be effective as a therapeutic tool for the treatment of infections [8,9] and wound healing [10,11,12]. Since they are known to efficiently produce atomic and molecular species in both the ground electronic state (such as NO, the hydroxyl radical OH, and atomic oxygen) and vibrationally- or electronically-excited states (with internal energies ranging from 0.1 eV to beyond 10 eV), low temperature plasmas are capable of driving chemistry far from thermal equilibrium. Consequently, plasma has been applied successfully to wound healing [13,14], tissue regeneration [15,16], bacterial inactivation [8,17], and cancer treatment [18,19]. In addition, this occurs in the absence of the significant heating of tissue or organs.

Regarding the study reported here, the weakly-ionized plasma generated in atmospheric-pressure expansions has been adopted as the plasma source. Often referred to as a cold atmospheric plasma jet (CAPJ), these plasmas can be produced in inexpensive, portable devices. Of greater importance are the atomic and molecular metastables generated in atmospheric pressure and rare gas jets that subsequently interact with room air. This results in the formation of the reactive atoms and molecules mentioned above, as well as other species that are expected to be lethal to viruses and bacteria. Since atmospheric pressure plasma expansions are also suitable for direct application to the tissue, several groups have shown that the CAPJ is effective in reducing bacterial loads and treating diverse infections [17,20]. Similarly, the effectiveness of the CAPJ in treating a variety of chronic wounds in the absence of side effects has been demonstrated over the past several years [11,21,22]. Examples include venous, arterial, pressure, and traumatic ulcers [13,23,24]. Increasing cutaneous microcirculation, keratinocyte and fibroblasts proliferation, monocyte stimulation, and cell migration [25,26] are among the mechanisms identified as responsible for wound healing. For the diabetic foot, plasma jets have been demonstrated to reduce bacterial load, enhance wound healing, and reduce pain for patients (relative to the control group), while dispensing with side effects, such as thermal damage [17,27]. For the skin wound model, the plasma treatment has been found to effectively decrease the wound size and promote re-epithelialization [20]. Increased expression of wound healing-related factors, such as VEGF, FGF, HBEGF, and IL-6 has also been demonstrated after the plasma treatment [28].

Regarding *C. albicans* infections, in vitro [29] and in vivo [30] studies have been reported for the inhibitory effect of CAPJ on the pathogen. Park et al. reported the effectiveness of microplasma jet arrays in the treatment of fungal keratitis in rabbits infected by *C. albicans* [10]. Although candidiasis is responsible for the significant increase in the morbidity and mortality of DM patients in recent years [2], the potential impact of atmospheric pressure plasma on *C. albicans* skin wound infections in patients with DM has not yet been investigated. Herein, we report on the development of a CAPJ system and its effect on the healing of diabetic skin wounds infected with *C. albicans*. Experiments demonstrate conclusively that low temperature plasma jets significantly reduced fungal infections and promoted skin wound healing, thus serving as an external (non-contact) biological stimulator. Infected wounds in rats were reduced in size by a factor of three, relative to the control group, by Day 10 following wound formation and no indications of thermal damage were observed when the wounds were treated with plasma. An in vitro experiment for the effect of CAPJ on *C. albicans* disinfection was also conducted.

## 2. Materials and Methods

### 2.1. Fabrication of CAPJ System

**Figure 1:**
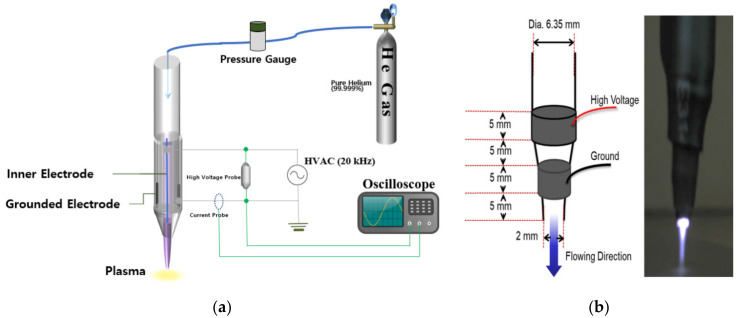
The plasma jet system used in this study. (**a**) Schematic diagram of the plasma jet system; (**b**) the inner structure and the gross photo of the single jet device.

An atmospheric pressure plasma jet system with a structure similar to what was described previously [31] was constructed and tested. Briefly, a single jet device with a 2 mm diameter (i.d.) aperture was fabricated within a block silicone polymer (Figure 1a). The device was designed to have the shape of a pen (cf. Figure 1b), which is convenient for both animal studies and clinical therapeutics. Throughout these experiments, the research grade He (99.999%) served as the carrier gas, the backing pressure was 800 Torr, and the plasma was generated within the cylindrical device by a power supply producing a 20 kHz sinusoidal voltage waveform with a RMS value of 0.88–1.06 kV. Both the internal (driven) and ground electrodes had widths of 5 mm and the separation between the two electrodes was also 5 mm. The operation of the plasma device is shown in Supplementary Video S1.

### 2.2. Characterization of Plasma Jet

Emission spectra produced by the plasma jet system were recorded, end-on to the jet, in the 200–800 nm wavelength region. With the plasma jet expanding into room air, spectra were acquired by a 0.75 m spectrometer with an 1800 lines/mm holographic grating and a diode array at the exit plane A collimating lens was also inserted into the beam path for collimation of the jet fluorescence. The effect of plasma on the surface modification of a glass slide was observed by applying the plasma jet to the surface of the glass for 5 s. Following exposure, the hydrophilicity of the glass surface of the slide glass was measured by the contact angle of a water drop (ventral view), and the result was compared with the control (no plasma treatment).

### 2.3. C. albicans and E. coli Culture and Plasma Treatment

To investigate the effectiveness of the plasma jet system on the sterilization of bacteria and fungi, *Escherichia coli* *(E. coli)* and *C. albicans* were cultured and treated in vitro. After *E. coli* was cultured at 37 °C for 24 h using a Luria–Bertani agar, a colony was transferred to 5 mL of nutrient broth. The optical density at 600 nm was measured, then 100 mL of nutrient broth was inoculated with the pre-culture corresponding to an optical density of 0.07. The bacteria were harvested by centrifugation and resuspended in PBS to a concentration of 10^7^ CFU/mL. After 100 μL of the bacterial suspension was plated on a Luria–Bertani agar surface and left to dry for 30 min, CAPJ was applied at a distance of 3 mm between the surface of the sample and the plasma jet for 30 s, 1, 3, and 5 min. *C. albicans* was cultured in sterile saline and subsequently the suspension was diluted by a factor of 100. After 100 μL of the suspension was applied to a Sabouraud dextrose agar-chloramphenicol (SDA-C) plate (Hangang, Seoul, Korea), macroscopic colonies grown after 24 h of incubation were treated with the plasma jet for 30 s, 1, 3, and 5 min.

### 2.4. Diabetic Rat Skin Wound Fungal Infection Model

Ten adult male Sprague–Dawley rats weighing between 300 and 400 g (aged between 10 and 12 weeks) were prepared for the animal study. The rats were subdivided into five time point groups (Days 1, 3, 5, 7, and 10), containing two animals in each group. The animals were housed separately under laboratory conditions and maintained under 12 h light–12 h dark cycles at a controlled temperature, with water and food made available *ad libitum*. After stabilization and acclimation of the rats for 7 days, diabetes was induced in the rats by an intraperitoneal injection of Streptozocin (Merck Korea, Seoul, Korea) with a dosage of 50 mg/kg for two successive days (Days −10~−9). Blood glucose was extracted at the tail vein and the level was checked 48−72 h after the second injection. When the glucose level was more than 200 mg/dL after 6 days (Days −4~−3), the rat was diagnosed as diabetic. The next day (1 week after the start of the diabetes induction, i.e., Day −3), the skin wound was made in the DM rat. After the rat was anaesthetized by an intraperitoneal injection of zoletil (virbac, 300 mg/kg), its dorsal hair was removed using an electric hair clipper and asepsia was performed with a povidone solution (Povidone-Iodine 10% Swabstick, Firson Co., Cheonan, Korea). Two square full-thickness dorsal skin wounds (1.2 × 1.2 cm in area) were made in the exposed dorsal skin for each rat with a dermatome by the method described by Suzuki et al. [32]. The distance between the two wounds were kept 2~3 cm, in order to minimize the effect of plasma to the control wound. Both wounds were infected with 100 μL of the suspension of *C. albicans* (5 × 10^7^ CFU/mL) each for two consecutive days following the dermal incisions (Days −2 and −1). The animal study was started the next day (Day 0), while one wound was treated by the CAPJ, the other one was not treated by plasma in order to serve as a control.

### 2.5. Animal Study

**Figure 2:**
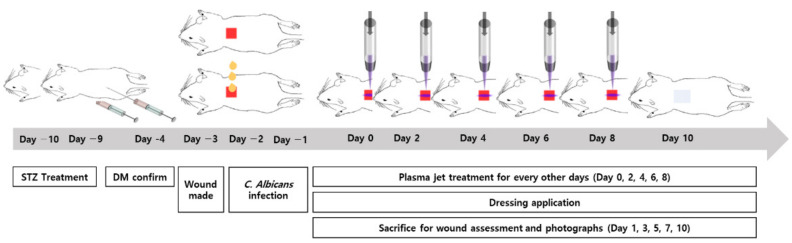
Timeline of *C. albicans* infected diabetic wound rat modeling and plasma jet treatment.

**Figure 3:**
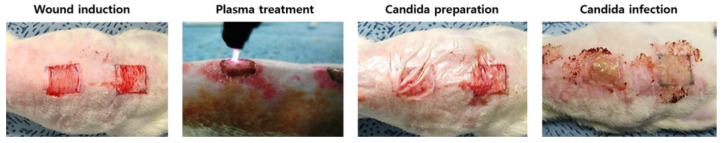
Representative gross photographs of the procedures.

After the skin wound was made and the infection was induced in the diabetic rat model, one wound in each rat was treated by plasma every other day (at Days 0, 2, 4, 6, and 8). Plasma was applied to the wound for 3 min (for 1 day for Day 1 group, 2 days for Day 3 group, 3 days for Day 5 group, 4 days for Day 7 group, and 5 days in total for Day 10 group, respectively) with the distance between the plasma and the wound surface maintained at 3 mm. The first treatment was applied 1 day after the *C. albicans* infection (Day 0), but not the next day (Day 1). However, two rats were sacrificed on Day 1 and immediately evaluated by the wound size and histological analysis. Eight rats were subjected to the plasma treatment for the second time on Day 2, and two rats among them were sacrificed the following day (Day 3). Therefore, five identical plasma treatments were performed on Days 0, 2, 4, 6, and 8 for the final two rats that were sacrificed at Day 10. Throughout the study, a wet gauze dressing using a saline-soaked gauze and a skin tape was applied to all wounds in the same manner once a day. Eschars formatted in the wound area were not removed to avoid any secondary injuries. The timeline and the representative gross photographs of the animal study are depicted in Figure 2 and Figure 3, respectively.

### 2.6. Wound Healing Analysis

The extent of the skin wound was determined through optical measurements of the wound area at Days 1, 3, 5, 7, and 10 (after the plasma treatment at Days 0, 2, 4, 6, and 8, respectively), on a living rat 6 h after the plasma treatment. The wound size was measured with an industrial-grade, charge-coupled device (CCD) camera and INNERVIEW 2.0 software. Moreover, the wound healing ratio was calculated by dividing the regenerated area (original size–wound size) by the original size of the wound (1.2 × 1.2 cm). The number of samples evaluated were 10 rats (10 plasma wounds, 10 control wounds) for Day 1, 8 rats for Day 3, 6 rats for Day 5, 4 rats for Day 7, and 2 rats for Day 10, respectively. The average values and their standard deviation were calculated for all measurements.

### 2.7. Histological Examination

Each day animals were sacrificed (at Days 1, 3, 5, 7, and 10), the harvested wound samples were fixed in a 10% buffered formalin solution, and specimens were embedded in a paraffin block and sectioned at 5 µm thickness. Two samples were included in all the subgroups of the time period (Days 1, 3, 5, 7, and 10) for both the control and plasma groups. The samples were stained with hematoxylin and eosin (H & E), Masson’s trichrome (MT) or periodic acid-Schiff (PAS). Epidermis, dermis, and subcutaneous layers were observed in coronal sections by optical microscopy (Eclipse 80i microscope, Nikon, Tokyo, Japan). Acute inflammation, fungal infection, granulation tissue, collagen deposition, blood clot or eschar formation, epidermis formation, keratin layer formation, and neovascularization were checked and recorded for each specimen.

### 2.8. Statistical Analysis

Statistical analysis of the data was performed with IBM SPSS Statistics software, version 20 (IBM Corporation, Armonk, NY, USA). Since the Shapiro-Wilk test rejected our normal distribution hypothesis for a 5% significance level, a nonparametric Wilcoxon signed rank test was selected for the analysis to compare the wound sizes between the two groups. Statistical significance was set at *p* < 0.05.

## 3. Results

### 3.1. Plasma Jet Characterization

Emission spectra of the He/plasma jet revealed the existence of several expected atomic and molecular species, such as O, OH, N_2_, and N_2_^+^, all of which have well-known bands and vibrational or electronic transitions in the 200–800 nm region. Dominant spectral signatures included the (v’,v”) = (0,0) and (0,1) transitions of the N_2_^+^ (B–X) band at 391.4 and 427.8 nm, respectively, the A–X band of OH lying near ~308 nm, and the (0,v” = 0–2) transitions of the C-B band of neutral N_2_ at 337.1, 357.7, and 380.5 nm, respectively [10]. In comparing the hydrophilicity of glass surface (untreated), as compared to the plasma-treated side, a significant difference was observed (Supplementary Figure S1). Specifically, the water contact angle for the control (untreated side of the glass) was 58.4° (58 ± 8), while for the plasma side that fell to 6.4° (6 ± 1), thus demonstrating that the exposure of the glass surface to the plasma jet renders the surface hydrophilic.

### 3.2. In Vitro Analysis of Plasma Treatment on C. albicans and E. coli

**Figure 4:**
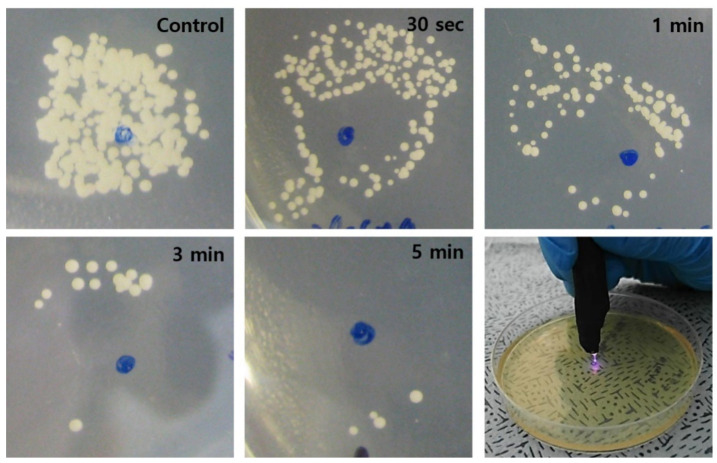
Photographs showing the time-dependent fungicidal (*C. albicans*) effect of the plasma jet. As the plasma treatment time increased, the area of complete sterilization (area without fungus) increased accordingly. Scale bars = 5 mm.

Supplementary Figure S2 shows representative optical micrographs of an *E. coli* culture as it is exposed to the plasma jet for increasing periods of the treatment. On the plasma-treated area of the agar plate, virtually no bacteria can be seen immediately after the plasma treatment, in the area of the surface that is directly treated. However, as the treatment time increased, the bacteria in the region lying outside the area treated directly by the plasma also decreased significantly in number. Similarly, the in vitro treatment of *C. albicans* with low temperature plasma, shown in Figure 4, resulted in the rapid disappearance of the fungus on the region of the SDA-C plate surface corresponding to the cross-sectional area of the plasma jet. As observed for *E. coli*, increasing the plasma exposure time again resulted in the vanishing of *C. albicans* from the area of the plate that was not exposed directly to the jet. These results are consistent with the conclusions of a previous study [33] that identified the diffusion of molecular radicals and electronically-excited species along the coordinate orthogonal to the axis of the plasma jet. The transverse diffusion of these reactive species, created at the interface of the He plasma with room air, extends the area of impact of the plasma well beyond the cross-sectional area of the jet.

### 3.3. Effect of CAPJ Treatment on C. albicans Infected Skin Wound

**Figure 5:**
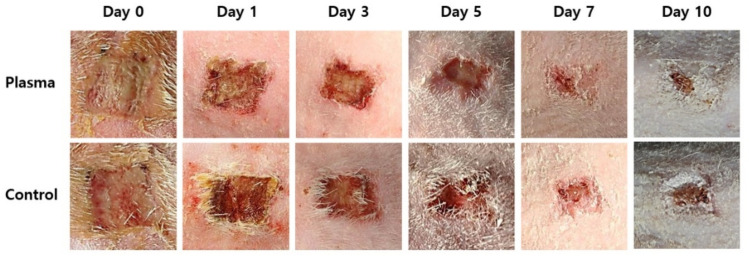
Representative gross photos of wound healing and closure in the plasma-treated and control groups by time.

**Figure 6:**
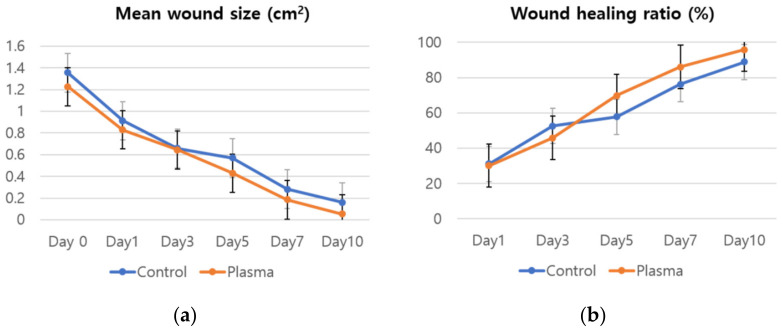
(**a**) Mean wound size and (**b**) wound healing ratio of the plasma-treated and control groups by time.

The mean weight of the rats before the STZ treatment was 372 ± 13.6 g with the mean glucose level of 97.7 ± 10.6 mg/dl. The values on the wound induction day were 331.1 ± 12.7 g and 314.3 ± 67.6 mg/dl, respectively, while the mean weight of the rats was 288.2 ± 23 g on the day the plasma treatment started. Figure 5 shows the representative gross photos of wounds with *C. albicans* infection in the diabetic rat model, and identifies the treatment time associated with each image. The defect size decreased as time passed and the effect is considerably more pronounced in the plasma-treated group, as compared to the control. The mean size of the dorsal skin wound and wound healing ratio, arranged according to the exposure time and treatment, are depicted in Figure 6. In the control group, the average wound sizes were 0.91, 0.66, 0.57, 0.28, and 0.16 cm^2^ at Days 1, 3, 5, 7, and 10, respectively. The average wound size in the plasma-treated group were 0.83, 0.64, 0.43, 0.18, and 0.05 cm^2^ at Days 1, 3, 5, 7, and 10, respectively. However, no significant difference was found in the statistical analysis between the two groups (*p*-values 0.215 for Day 0, 0.092 for Day 1, 0.514 for Day 3, 0.144 for Day 5, 0.573 for Day 7, and 0.204 for Day 10). The calculated wound healing ratio in the control group was 31.18, 52.63, 57.74, 76.2, and 88.94% at Days 1, 3, 5, 7, and 10, respectively, while the corresponding ratio was 30.18, 45.87, 69.77, 86.06, and 95.79%, respectively in the plasma-treated group. Compared to the control group, the plasma-treated wound showed accelerated wound closure, particularly after Day 5.

**Figure 7:**
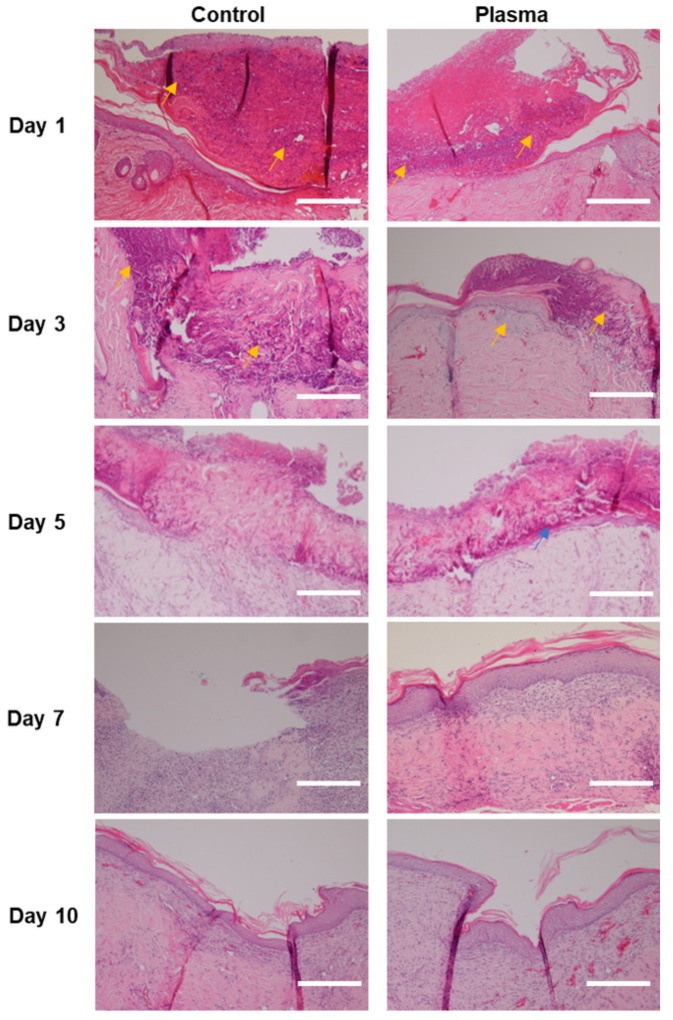
Representative histological images stained with hematoxylin and eosin of the skin wound of diabetic rat infected with *C. albicans* according to time. Faster tissue regeneration and less infiltration of inflammatory cells (yellow arrow) are noted in the plasma group compared to the control. Formation of keratin pearl was noted in the plasma-treated group at Day 5 (blue arrow). Epithelium as well as stroma are still damaged in the control group at Day 7, compared to the plasma-treated group, in which they are almost recovered. Original magnification × 20; scale bars = 250 um.

An histologic evaluation of the tissue samples revealed the infiltration of lymphocytes and neutrophils in the epidermis and dermis layers of the wound in both groups (Figure 7). However, the plasma-treated group showed less inflammatory cells per HPF for all of the time period, as compared to the control. Furthermore, tissue damage remained in the control group at Days 3 and 5 with little evidence of healing, which contrasted with the plasma-treated group, in which more rapid healing and the appearance of keratin pearl was observed. A defected wound healing was still noted in Day 7 of the control group, whereas an almost complete regeneration of tissue occurred at Day 7 in the plasma group, as evidenced by the clear and healthy distinction between the epidermal, dermal, and subcutaneous layers. At Day 10, an immature re-epithelialization was found in the control group, while an almost complete healing of the skin wound with neovascularization was observed in the plasma group. The plasma jet-treated wounds did not reveal any thermal damage in the subepithelial region at any point in this study.

**Figure 8:**
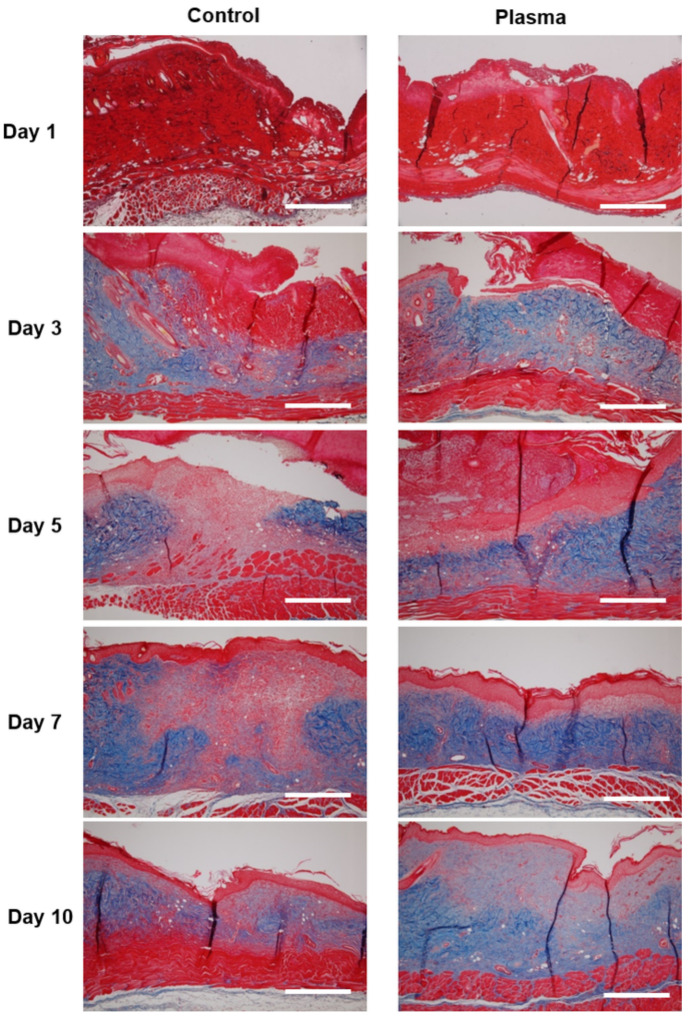
Representative histological analysis stained with Masson’s trichrome at the indicated time points. Collagen deposition (blue) was denser in the plasma-treated group after Day 3, as compared to the control. Original magnification × 10; scale bars = 500 um.

**Figure 9:**
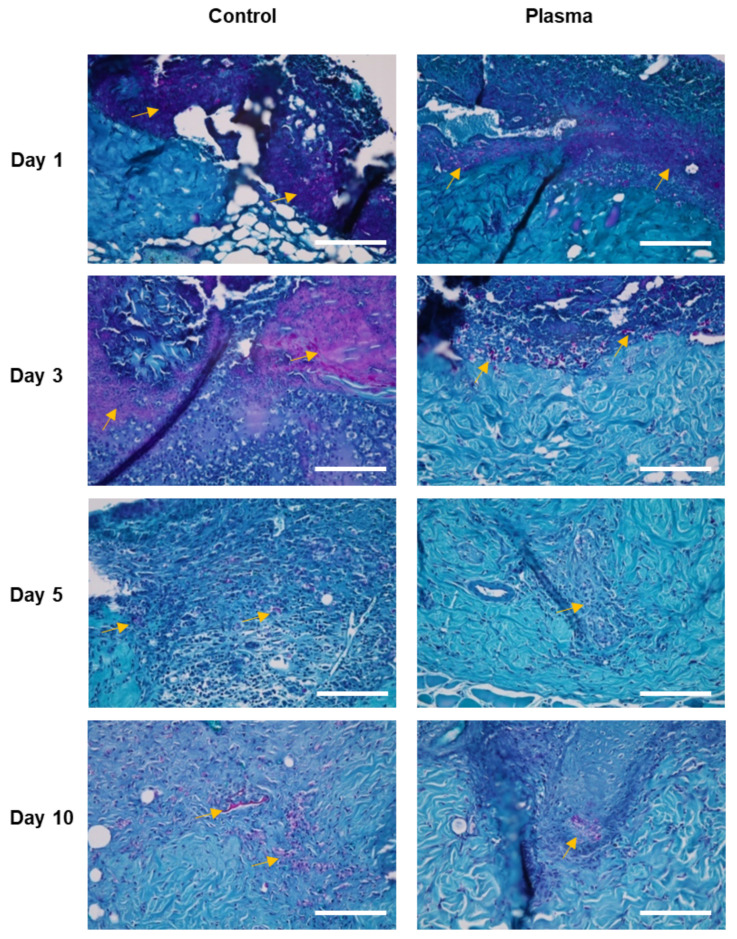
Representative histological images of the skin wound of diabetic rat infected with *C. albicans* stained with periodic acid-Schiff. Fungal infection (yellow arrows, cell stained with magenta) was more prominent in the control group compared to the plasma-treated group. Original magnification × 40; scale bars = 200 um.

The histologic morphology of the MT stained slides revealed abundant collagen growth and deposition in the plasma-treated group, as compared to the control (Figure 8). The collagen arrangement for the plasma group was also significantly more dense and spatially-uniform than observed for the control. Moreover, the regeneration of the dermis was more prominent in the plasma group, as compared to the control. Finally, a prominent fungal infection infiltrating into the dermis was noted in the PAS stain of the control group (Figure 9). Although the epidermis layer was healed with sufficient time, a fungal infection was observed in the dermis layer of the control specimens until Day 10. However, the plasma-treated skin revealed less fungal cells per HPF (as indicated by the PAS stain).

## 4. Discussion

Low temperature plasma has been demonstrated to have a significant potential for use in a wide range of modern clinical practice. Specifically, it has recently become one of the innovative approaches to wound healing treatment. Wound healing is a complex and dynamic process that remains one of the major challenges in the biomedical field. The process requires the integration of biological and molecular events, such as cell migration, proliferation, extracellular matrix deposition, and remodeling [34,35]. Re-epithelization, one aspect of the regeneration process of the skin, recruits the cellular and humoral components by epidermal and dermal interactions via signal molecules [36]. Moreover, extensive studies have reported decreased bacterial load and improved wound healing with the plasma treatment [1,11,12,31,36,37]. By producing reactive species, low temperature plasma is known to accelerate the tissue repair process and reduce microbial load without negative effects on normal tissues [18].

Although the plasma treatment is known to promote the repair and regeneration of dermal tissues, it can also be applied to the regeneration of deeply-embedded organs and structures by developing specific plasma devices [18]. Microplasmas, plasma confined to cavities with micron-scale dimensions, have been shown for more than two decades to produce various reactive species more efficient than conventional (macroscopic) plasma devices due to the higher electron number densities, electron temperatures, and operating pressures available with microplasma devices and arrays [12]. Arrays of plasma jets, as well as single electrode plasma jet devices, have also been widely investigated for use in medical applications [10,12,38,39,40].

While the underlying mechanism of low temperature plasma on wound healing is not fully understood, Kang et al. have reported that healing is enhanced by promoting the epithelial-to-mesenchymal transition and by activating the matrix metalloproteinase-9/urokinase-type plasminogen activator system [37]. Increased cell proliferation, which is the pivotal step in the wound healing process, facilitates granulation tissue formation and alleviates inflammation in cutaneous tissue, which results in accelerated wound closure [14]. Nastuta et al. have reported increased oxidative stress inside the living plasma-treated tissue, in which plasma-generated species, such as the OH radical, are presumed to be responsible [36]. At the same time, inflammation parameters were observed to remain at relatively low levels with the plasma treatment [36]. The authors also reported accelerated keratinocyte and fibrocyte responses after the plasma treatment. However, it must be mentioned that differing mechanisms are expected to be dominant in the healing of different wounds treated by low temperature plasma. For example, the primary mechanism for wound healing in second and third-degree burns was suggested to be enhanced angiogenesis [18]. 

The impact of plasma on human epidermal growth factor (hEGF) should be considered, as hEGF plays a significant role in cell growth and differentiation by binding to its receptor, epidermal growth factor receptor (EGFR). As opposed to the cancer treatment using a longer time of CAPJ treatment with higher oxidation degree, a low amount of modified amino acids by a short time of CAPJ treatment has been reported to cause minimal structural changes in hEGF and impact on the interaction with EGFR, favoring cell proliferation in wound healing while decontaminating the pathogens [41]. Increased glucose uptake in the skeletal muscle cells of diabetic rat by the reactive species generated by CAPJ has been reported by Kumar et al., which represents another underlying mechanism of fungicidal effect of plasma in diabetic subjects [42]. The changes of immune cells after plasma treatment should also be recognized, considering the major contribution of immune cells in wound healing. Bekeschus et al. have proven that the short term application of the atmospheric pressure argon plasma jet did not inhibit the proliferation nor activation of T cells [43].

Regarding diabetic foot ulcers in humans, a randomized clinical trial revealed that the low temperature plasma treatment of wounds significantly accelerated the healing process, as compared to the control [1]. In the diabetic rat model, Fathollah et al. reported a significant wound contraction after plasma treatment (compared to control) [11]. The formation of an epidermis layer, neovascularization, and cell proliferation was observed after plasma treatment, and the release of TGF-β1 from cells were also confirmed in this study [11]. The effect of plasma-generated reactive species on *C. albicans* disinfection has been demonstrated by Souza et al. [44]. Using aminoguanidine, the activity of neutrophil reactive oxygen (NOX2) and, subsequently, the microbicidal activity of neutrophil itself both increased. In accordance with these findings, our study has demonstrated the effect of low temperature plasma in enhancing skin wound healing of diabetic rat skin wounds infected by *C. albicans*. These findings confirm the potential of low temperature plasma treatment as a therapeutic choice for fungal wound infections in diabetic patients.

In our previous study, microplasma jet arrays were observed to provide a therapeutic effect with respect to fungus (*C. albicans*), as well as bacteria (*E. coli*), both in vitro and in vivo [12]. Furthermore, the effect on *C. albicans* infections of rabbit eyes was demonstrated [10]. Compared to single-jet devices, arrays of microplasma jets offer several advantages over a single plasma source, including the ability to address specific jets, larger treatment areas and, through the introduction of differing gas mixtures in specific jets in the arrays, selective generation of medically-relevant reactive species [12]. Microplasma jet arrays were also tested for the treatment of diabetic skin wounds in the present study but no significant difference in wound healing was observed (as compared to control) when both types of low temperature plasma jet devices employed an He-only gas flow. The single-jet device described earlier demonstrated a significant decrease in the size of diabetic rat model wounds and increased re-epithelialization through collagen arrangement, as compared to wounds that were not treated with the plasma. The histologic morphology also showed faster recovery of the infected wound in the plasma-treated group, as compared to control. The CAPJ treatment was applied to the diabetic wound for a 3 min time period, based on the in vitro study that revealed to sterilize almost all of the pathogens in the plasma-treated area in a 3 min group (yet above the treatment area in a 5 min group).

Our hypothesis is that the reactive species generated from the interaction of the low temperature plasma jet with room air deactivates *C. albicans* and reverses its adverse impact on healing of the diabetic skin wound. It has been reported that several diatomic and triatomic molecules (such as NO and O_3_, respectively), produced by the interaction of low-temperature plasma and atmospheric air, are capable of deactivating microorganisms [12,45]. The reactive species produced by the interaction of N_2_^+^ ions and background oxygen or nitrogen are thought to be responsible for lipid peroxidation of bacteria membranes for the eradication of *E. coli* [46]. However, in order to elucidate the underlying molecular mechanisms responsible for the accelerated healing of wounds infected with *C. albicans*, further molecular studies will be required. The limitation of this study includes the fact that a low number of cases was included for each group. Future studies with a larger sample size are needed. The clinical application of this study includes an introduction of a novel therapeutic approach to the treatment of infected diabetic skin wounds, with high efficiency but less morbidity. However, further human studies will be necessary in order for the low temperature plasma to be applied in routine clinical practice.

## 5. Conclusions

As an addition to the application spectrum of low temperature plasma in the medical field, we report here on the positive impact of He plasmas on the healing of diabetic skin wounds infected by *C. albicans* in a rat model. Without evidence of a negative effect on the surrounding tissue, the plasma showed a reduction of fungal infection and indications of accelerated skin wound healing by serving as a non-contact external biological stimulator. With further studies on humans, low temperature plasma jets are likely to be a treatment option that will lead to a more effective but less invasive treatment, thereby reducing the financial burden of skin wound infections on diabetic patients.

## Data Availability

The data presented in this study are available on request from the corresponding author.

## Supplementary Materials

The following are available online at https://www.mdpi.com/article/10.3390/biomedicines10010027/s1. Video S1: Video representing the actual operation of the plasma jet device used in this study. Figure S1: Above view and central view showing the hydrophilicity of water at the slide surface treated by plasma (for 5 s) and control (no plasma treatment). Figure S2: Photographs showing the time-dependent bactericidal (*E. coli*) effect of the plasma jet.

## References

[1] Mirpour S., Fathollah S., Mansouri P., Larijani B., Ghoranneviss M., Mohajeri Tehrani M., Amini M.R. (2020). Cold atmospheric plasma as an effective method to treat diabetic foot ulcers: A randomized clinical trial. Sci. Rep..

[2] Rodrigues C.F., Rodrigues M.E., Henriques M. (2019). Candida sp. Infections in Patients with Diabetes Mellitus. J. Clin. Med..

[3] Wilson R.M., Reeves W.G. (1986). Neutrophil phagocytosis and killing in insulin-dependent diabetes. Clin. Exp. Immunol..

[4] Costa S.F., Marinho I., Araújo E.A.P., Manrique A.E.I., Medeiros E.A.S., Levin A.S. (2000). Nosocomial fungaemia: A 2-year prospective study. J. Hosp. Infect..

[5] Abelson J.A., Moore T., Bruckner D., Deville J., Nielsen K. (2005). Frequency of Fungemia in Hospitalized Pediatric Inpatients Over 11 Years at a Tertiary Care Institution. Pediatrics.

[6] Andrews K.L., Houdek M.T., Kiemele L.J. (2015). Wound management of chronic diabetic foot ulcers: From the basics to regenerative medicine. Appl. Environ. Microbiol..

[7] Singh N., Armstrong D.G., Lipsky B.A. (2005). Preventing foot ulcers in patients with diabetes. JAMA.

[8] Yan D., Malyavko A., Wang Q., Ostrikov K., Sherman J.H., Keidar M. (2021). Multi-Modal Biological Destruction by Cold Atmospheric Plasma: Capability and Mechanism. Biomedicines.

[9] Sakudo A., Yagyu Y., Onodera T. (2019). Disinfection and Sterilization Using Plasma Technology: Fundamentals and Future Perspectives for Biological Applications. Int. J. Mol. Sci..

[10] Park H.J., Kim S.H., Ju H.W., Lee H., Lee Y., Park S., Yang H., Park S.J., Eden J.G., Yang J. (2018). Microplasma Jet Arrays as a Therapeutic Choice for Fungal Keratitis. Sci. Rep..

[11] Fathollah S., Mirpour S., Mansouri P., Dehpour A.R., Ghoranneviss M., Rahimi N., Safaie Naraghi Z., Chalangari R., Chalangari K.M. (2016). Investigation on the effects of the atmospheric pressure plasma on wound healing in diabetic rats. Sci. Rep..

[12] Lee O.J., Ju H.W., Khang G., Sun P.P., Rivera J., Cho J.H., Park S.J., Eden J.G., Park C.H. (2016). An experimental burn wound-healing study of non-thermal atmospheric pressure microplasma jet arrays. J. Tissue Eng. Regen. Med..

[13] Chuangsuwanich A., Assadamongkol T., Boonyawan D. (2016). The Healing Effect of Low-Temperature Atmospheric-Pressure Plasma in Pressure Ulcer: A Randomized Controlled Trial. Int. J. Low. Extrem. Wounds.

[14] Lou B.S., Hsieh J.H., Chen C.M., Hou C.W., Wu H.Y., Chou P.Y., Lai C.H., Lee J.W. (2020). Helium/Argon-Generated Cold Atmospheric Plasma Facilitates Cutaneous Wound Healing. Front. Bioeng. Biotechnol..

[15] Arndt S., Unger P., Bosserhoff A.-K., Berneburg M., Karrer S. (2021). The Anti-Fibrotic Effect of Cold Atmospheric Plasma on Localized Scleroderma In Vitro and In Vivo. Biomedicines.

[16] Choi J.W., Kang S.U., Kim Y.E., Park J.K., Yang S.S., Kim Y.S., Lee Y.S., Lee Y., Kim C.H. (2016). Novel Therapeutic Effects of Non-thermal atmospheric pressure plasma for Muscle Regeneration and Differentiation. Sci. Rep..

[17] Isbary G., Morfill G., Schmidt H.U., Georgi M., Ramrath K., Heinlin J., Karrer S., Landthaler M., Shimizu T., Steffes B. (2010). A first prospective randomized controlled trial to decrease bacterial load using cold atmospheric argon plasma on chronic wounds in patients. Br. J. Dermatol..

[18] Brany D., Dvorska D., Halasova E., Skovierova H. (2020). Cold Atmospheric Plasma: A Powerful Tool for Modern Medicine. Int. J. Mol. Sci..

[19] Izadjoo M., Zack S., Kim H., Skiba J. (2018). Medical applications of cold atmospheric plasma: State of the science. J. Wound Care.

[20] Bernhardt T., Semmler M.L., Schäfer M., Bekeschus S., Emmert S., Boeckmann L. (2019). Plasma Medicine: Applications of Cold Atmospheric Pressure Plasma in Dermatology. Oxid. Med. Cell. Longev..

[21] Cheng K.Y., Lin Z.H., Cheng Y.P., Chiu H.Y., Yeh N.L., Wu T.K., Wu J.S. (2018). Wound Healing in Streptozotocin-Induced Diabetic Rats Using Atmospheric-Pressure Argon Plasma Jet. Sci. Rep..

[22] Li D., Li G., Li J., Liu Z.-Q., Zhang X., Zhang Y., Li H.-P. (2019). Promotion of Wound Healing of Genetic Diabetic Mice Treated by a Cold Atmospheric Plasma Jet. IEEE Trans. Plasma Sci..

[23] Isbary G., Heinlin J., Shimizu T., Zimmermann J.L., Morfill G., Schmidt H.-U., Monetti R., Steffes B., Bunk W., Li Y. (2012). Successful and safe use of 2 min cold atmospheric argon plasma in chronic wounds: Results of a randomized controlled trial. Br. J. Dermatol..

[24] Brehmer F., Haenssle H.A., Daeschlein G., Ahmed R., Pfeiffer S., Görlitz A., Simon D., Schön M.P., Wandke D., Emmert S. (2015). Alleviation of chronic venous leg ulcers with a hand-held dielectric barrier discharge plasma generator (PlasmaDerm^®^ VU-2010): Results of a monocentric, two-armed, open, prospective, randomized and controlled trial (NCT01415622). J. Eur. Acad. Dermatol. Venereol..

[25] von Woedtke T., Reuter S., Masur K., Weltmann K.D. (2013). Plasmas for medicine. Phys. Rep..

[26] Xiong Z. (2018). Cold Atmospheric Pressure Plasmas (CAPs) for Skin Wound Healing. Plasma Medicine—Concepts and Clinical Applications.

[27] Heinlin J., Morfill G., Landthaler M., Stolz W., Isbary G., Zimmermann J.L., Shimizu T., Karrer S. (2010). Plasma medicine: Possible applications in dermatology. J. Dtsch. Dermatol. Ges..

[28] Guo H., Li P., Li H.P., Ge N., Bao C.Y. (2016). In situ measurement of the two-dimensional temperature field of a dual-jet direct-current arc plasma. Rev. Sci. Instrum..

[29] Maisch T., Shimizu T., Isbary G., Heinlin J., Karrer S., Klämpfl T.G., Li Y.-F., Morfill G., Zimmermann J.L. (2012). Contact-free inactivation of Candida albicans biofilms by cold atmospheric air plasma. Appl. Environ. Microbiol..

[30] Borges A.C., Lima G.M.G., Nishime T.M.C., Gontijo A.V.L., Kostov K.G., Koga-Ito C.Y. (2018). Amplitude-modulated cold atmospheric pressure plasma jet for treatment of oral candidiasis: In vivo study. PLoS ONE.

[31] Park C.H., Lee J.S., Kim J.H., Kim D.-K., Lee O.J., Ju H.W., Moon B.M., Cho J.H., Kim M.H., Sun P.P. (2014). Wound healing with nonthermal microplasma jets generated in arrays of hourglass microcavity devices. J. Phys. D Appl. Phys..

[32] Suzuki T., Sawada Y. (1989). A dermatome for experiments with small animals. Eur. J. Plast. Surg..

[33] Sun P.P., Chen H.L., Park S.J., Eden J.G., Liu D.X., Kong M.G. (2015). Off-axis chemical crosstalk in an atmospheric pressure microplasma jet array. J. Phys. D Appl. Phys..

[34] Ravanti L., Kahari V.M. (2000). Matrix metalloproteinases in wound repair (review). Int. J. Mol. Med..

[35] Wu A.S., Kalghatgi S., Dobrynin D., Sensenig R., Cerchar E., Podolsky E., Dulaimi E., Paff M., Wasko K., Arjunan K.P. (2013). Porcine intact and wounded skin responses to atmospheric nonthermal plasma. J. Surg. Res..

[36] Nastuta A.V., Topala I., Grigoras C., Pohoata V., Popa G. (2011). Stimulation of wound healing by helium atmospheric pressure plasma treatment. J. Phys. D Appl. Phys..

[37] Kang S.U., Choi J.W., Chang J.W., Kim K.I., Kim Y.S., Park J.K., Kim Y.E., Lee Y.S., Yang S.S., Kim C.H. (2017). N2 non-thermal atmospheric pressure plasma promotes wound healing in vitro and in vivo: Potential modulation of adhesion molecules and matrix metalloproteinase-9. Exp. Dermatol..

[38] Schmidt A., Bekeschus S., Wende K., Vollmar B., von Woedtke T. (2017). A cold plasma jet accelerates wound healing in a murine model of full-thickness skin wounds. Exp. Dermatol..

[39] Lu X., Jiang Z., Xiong Q., Tang Z., Pan Y. (2008). A single electrode room-temperature plasma jet device for biomedical applications. Appl. Phys. Lett..

[40] Sun P.P., Won J., Choo-Kang G., Li S., Chen W., Monroy G.L., Chaney E.J., Boppart S.A., Eden J.G., Nguyen T.H. (2021). Inactivation and sensitization of Pseudomonas aeruginosa by microplasma jet array for treating otitis media. NPJ. Biofilms Microbiomes.

[41] Yusupov M., Lackmann J.-W., Razzokov J., Kumar S., Stapelmann K., Bogaerts A. (2018). Impact of plasma oxidation on structural features of human epidermal growth factor. Plasma Process. Polym..

[42] Kumar N., Shaw P., Razzokov J., Yusupov M., Attri P., Uhm H.S., Choi E.H., Bogaerts A. (2018). Enhancement of cellular glucose uptake by reactive species: A promising approach for diabetes therapy. RSC Adv..

[43] Bekeschus S., Masur K., Kolata J., Wende K., Schmidt A., Bundscherer L., Barton A., Kramer A., Bröker B., Weltmann K.-D. (2013). Human Mononuclear Cell Survival and Proliferation is Modulated by Cold Atmospheric Plasma Jet. Plasma Process. Polym..

[44] de Souza Ferreira C., Pennacchi P.C., Araújo T.H., Taniwaki N.N., de Araújo Paula F.B., da Silveira Duarte S.M., Rodrigues M.R. (2016). Aminoguanidine treatment increased NOX2 response in diabetic rats: Improved phagocytosis and killing of Candida albicans by neutrophils. Eur. J. Pharmacol..

[45] Graves D.B. (2012). The emerging role of reactive oxygen and nitrogen species in redox biology and some implications for plasma applications to medicine and biology. J. Phys. D Appl. Phys..

[46] Brun P., Pathak S., Castagliuolo I., Palu G., Brun P., Zuin M., Cavazzana R., Martines E. (2014). Helium generated cold plasma finely regulates activation of human fibroblast-like primary cells. PLoS ONE.

