# Supplementary figures and images for: Treatment of Fungal-Infected Diabetic Wounds with Low Temperature Plasma
Source: Biomedicines. 2021 Dec 23;10(1):27. doi: 10.3390/biomedicines10010027 (PMC8773309; doi:10.3390/biomedicines10010027)

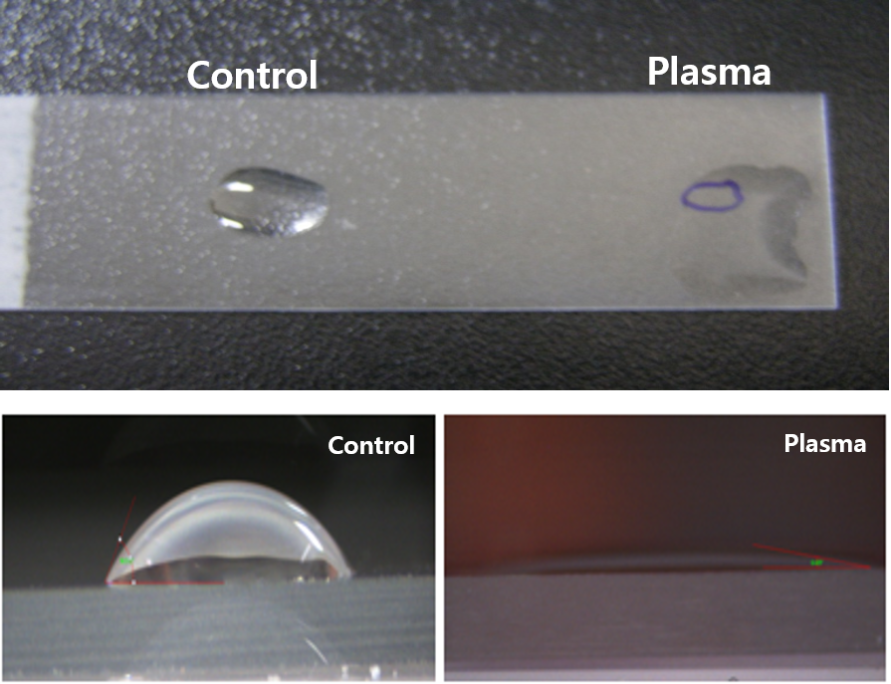

Supplement: Supplementary file 1 [file biomedicines-10-00027-s001.zip › biomedicines-1483255-supplementary/Supplementary Figure S1.tif]

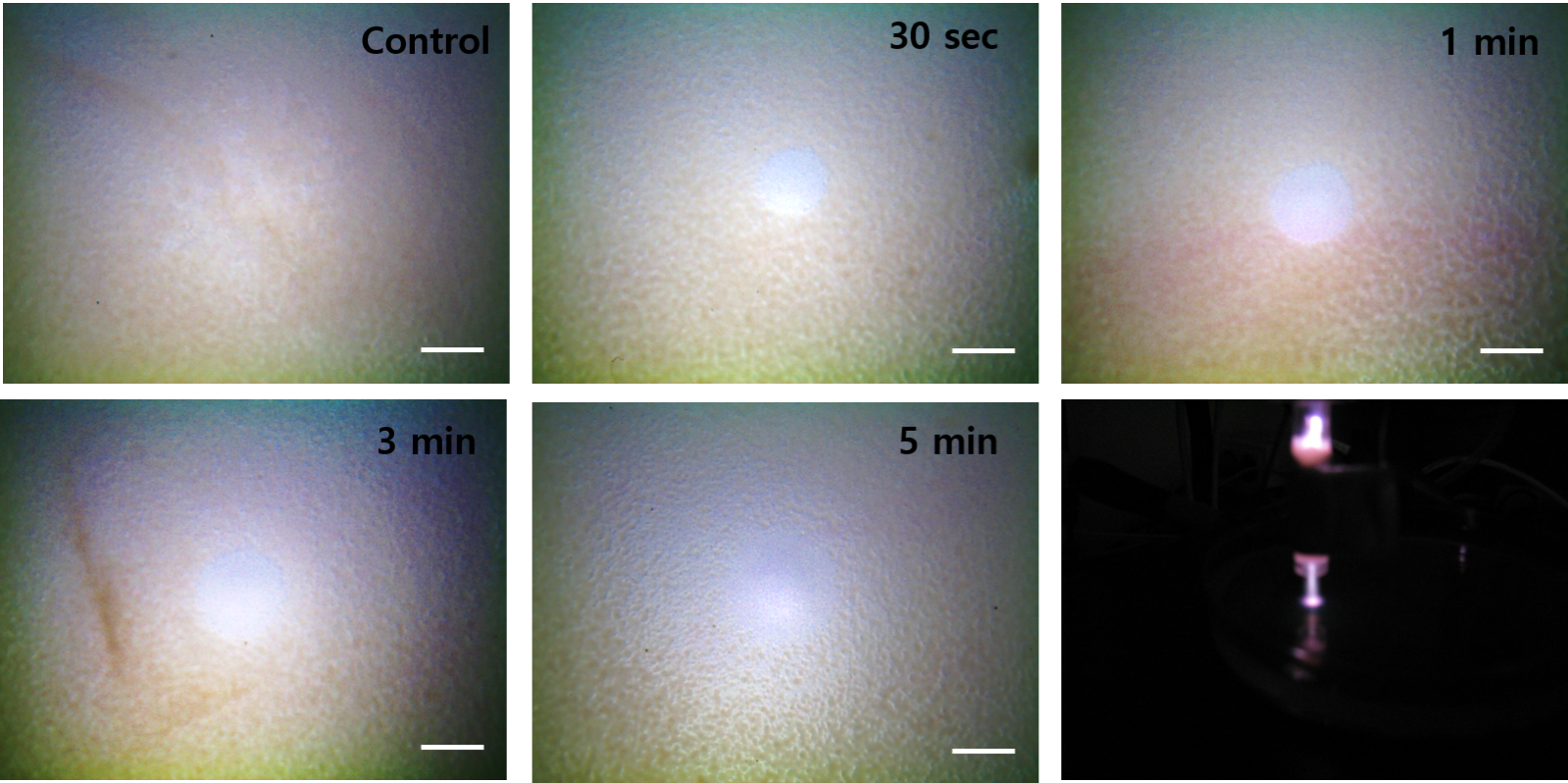

Supplement: Supplementary file 1 [file biomedicines-10-00027-s001.zip › biomedicines-1483255-supplementary/Supplementary Figure S2.tif]
